# Acute liver failure caused by *Amanita verna*: a case series and review of the literature

**DOI:** 10.1186/s12893-021-01434-6

**Published:** 2021-12-25

**Authors:** Jianlong Wu, Xueyi Gong, Zemin Hu, Qiang Sun

**Affiliations:** Department of General Surgery, Zhongshan Hospital, Sun Yat-Sen University, No. 2 Sunwen East Road, Shiqi District, Zhongshan, 528403 Guangdong People’s Republic of China

**Keywords:** Acute liver failure, *Amanita verna*, Liver transplantation, Survival rate, Therapy

## Abstract

**Background:**

*Amanita verna* is one of the most harmful wild fungi in China. *Amanita verna* poisoning occurs every year, and the mortality is as high as 50%. However, its clinical manifestations are complex and diverse.

**Case presentation:**

In March 2019, three patients took a large amount of Amanita, and one of them received liver transplantation in Zhongshan hospital, Sun Yat-sen University. All patients had vomiting and diarrhea 8–12 h after eating wild mushrooms (Amanita). The patients were initially diagnosed with Amanita poisoning. One case (case 3) was complicated and diagnosed as mushroom poisoning (fatal Amanita), toxic hepatitis, acute liver failure, toxic encephalopathy, hemorrhagic colitis, toxic myocarditis, disseminated intravascular coagulation (DIC) and pregnancy. The general clinical data of all patients were recorded, who received early treatment such as hemodialysis, artificial liver plasma exchange, hormone shock and anti-infection. One case (case 1) recovered smoothly after liver transplantation, and the indexes of liver, kidney, coagulation function and infection were improved. The other two cases died of intracerebral hemorrhage.

**Conclusion:**

Liver transplantation is an effective method for the treatment of acute liver failure caused by mushroom poisoning and can improve the survival rate of patients with toxic liver failure.

## Background

*Amanita verna* is the most toxic wild fungus in China. It is poisoned every year, and the mortality is as high as 50% [1]. Mushroom poisoning has become the most serious foodborne disease in China [2]. Amanita is distributed in Hebei, Jilin, Liaoning, Shenyang, Jiangsu, Fujian, Anhui, Shaanxi, Gansu, Hubei, Hunan, Shanxi, Guangxi, Guangdong, Sichuan, Yunnan, Tibet and other places in China. Its clinical manifestations are complex and diverse. Most patients take nausea, vomiting, abdominal pain, diarrhea and other gastrointestinal symptoms as the initial manifestation of poisoning [3]. According to clinical manifestations, mushroom poisoning can be divided into gastroenteritis type, multiple organ damage type (liver damage type, renal failure type), neuropsychiatric type and hemolytic type [4]. Among them, 95% of wild mushroom poisoning incidents were caused by Amanita [5]. Amanitin is the most important lethal toxin in Amanita. It is soluble in water, chemically stable, resistant to high temperature, acid and alkali [6]. After eating, it can be quickly absorbed into the liver by the digestive tract, combined with RNA polymer of hepatocytes, inhibit the production of mRNA, lead to hepatocyte necrosis and acute liver failure, and seriously endanger human health [7]. Therefore, how to identify fatal mushroom poisoning as soon as possible and treat it timely and effectively is a great challenge for medical staff.

In March 2019, three patients with Amanita poisoning were treated at the same time, and one of whom received liver transplantation. In this study, we analyzed the clinical data of these three patients and discussed the indications and timing of liver transplantation for liver failure caused by toxic mushroom poisoning.

## Case presentation

### Characteristics of three cases

Three patients had food poisoning when eating wild mushrooms (*Amanita verna*) at the same time. Among them, the patient of case 2 took mushrooms and soup, and the other two patients only took mushrooms. All patients developed vomiting and diarrhea 8–12 h after eating. After gastric lavage and rehydration in the local hospital, their condition deteriorated, and they were transferred to Zhongshan hospital, Sun Yat-sen University. Table 1 showed the characteristics of three patients at the time of admission. The patient was initially diagnosed as Amanita poisoning. Clinical diagnosis indicated that the patient of case 3 was Amanita poisoning, toxic hepatitis, acute liver failure, toxic encephalopathy, hemorrhagic colitis, toxic myocarditis, disseminated intravascular coagulation and pregnancy.

**Table 1:** Characteristics of three patients at the time of admission

Patients	1	2	3
Gender	Male	Male	Female
Age	43	43	34
Time of admission	March 4, 2019	March 4, 2019	The patient was admitted to the hospital on March 9, 2019, and was transferred to Zhongshan hospital, Sun Yat-sen University after coma at about 11 am
Cause of hospitalization	Nausea, vomiting and diarrhea occurred 18 h after eating wild mushrooms	Nausea, vomiting, and diarrhea occurred 18 h after eating wild mushrooms	Vomiting and diarrhea for 7 days, and vomiting for 1 day after eating mushrooms
Previous medical history	Good health, no special problems	Good health, no special problems	Good health. The patient was pregnant twice with one child in the second trimester
Mushroom intake	One bowl	Two bowls (about 20 Amanita) and a bowl of noodle soup (without Amanita)	Three bowls
Four vital signs	T: 37.4 °C, P: 93 times/min, R: 20 times/min, BP: 139/57 mmHg	T: 36.8 °C, P: 75 times/min, R: 16 times/min, BP: 126/76 mmHg	T: 36.8 °C, P: 101 times/min, R: 23 times/min, BP: 131/96 mmHg
Other physical examination items	Mind clear. The skin of the extremities and lips were dry. The abdomen was flat and soft. There was no muscle tension. There was no tenderness or rebound tenderness over entire abdomen. The frequency of bowel sound was 3 times/min	Mind clear. The skin and sclera were yellow, the abdomen was soft, the upper abdomen was tender, and there was no rebound pain. The liver was large, 2 cm below the ribs. There was no edema in the lower limbs, and the muscle strength and muscle tension of the limbs were normal	The patient was in a coma, has no stinging eyes, tingles and bends, and can produce a single tone. The examination was uncooperative, the pupils were symmetrical, the light reflex was slow, and the sclera and skin and mucosa were yellow. Abdominal eminence corresponds to gestational age. Old surgical scar can be seen in the right lower abdomen. The patient did not cooperate with examinations of abdominal tenderness and rebound pain. Bowel sounds are high, about 10 times per minute. Did not cooperate with muscle strength and muscle tension tests of limbs
White blood cells	19.24 × 10^9^/L	8.16 × 10^9^/L	13.31 × 10^9^/L
Percentage of neutrophils	95.4%	77.7%	–
Platelets	68 × 10^9^/L	–	48 × 10^9^/L
3P test	Negative (−)	Negative (−)	Positive (+)
d-dimer	1.13 mg/L	–	39.13 mg/L
ALT	126 U/L	39 U/L	571 U/L
AST	139 U/L	48 U/L	119 U/L
Blood ammonia	–	–	138.8 μmol/L
Prothrombin time and ratio	11.5 s; 0.96	–	16.9 s; 1.41
Fibrinogen	1.39 g/L	1.44 g/L	1.51 g/L
Total bilirubin	–	23.9 μmol/L	195.7 μmol/L
Cardiopulmonary	No abnormalities	–	–
Abdomen	There was gallbladder after meals. No obvious abnormalities in liver and spleen. The pancreas was unclear. No obvious abnormalities were found in kidneys and ureters	–	–

According to individual clinical indications, the treatment methods of each patient are continuously adjusted. Outcomes of each patient were shown in Table 2. The brief process and prognosis were as follows.

**Table 2:** Outcomes

Case	Diagnosis to treatment	Liver transplantation	Final Indicators	Outcome
1	March 4 to March 28	Yes	On March 28 ALT: 160 U/L, direct bilirubin: 19.6 μmol/L, total bilirubin: 42.1 μmol/L. Coagulation disorders have been corrected. The mean portal blood flow velocity and hepatic artery resistance index were normal. There was fluid in the right chest. No obvious effusion was found in the abdominal cavity	The patient recovered and was discharged
2	March 4 to March 26	NO	On March 20 The patient was mentally vague, unconscious, bilateral pleural effusion increased, pelvic effusion increased; cerebral hemorrhage and brain stem damage	Patient died
3	March 10 to May 15	NO	On May 5 The patient was in a coma, responded slowly to light without tongue extension. The muscle tension of the left limb was low, and the meningeal irritation sign was negative. The patient had massive hemorrhagic cerebral infarction and was still in coma after operation On May 15, the family requested to be discharged	Patient died

### Case 1

**Fig. 1:**
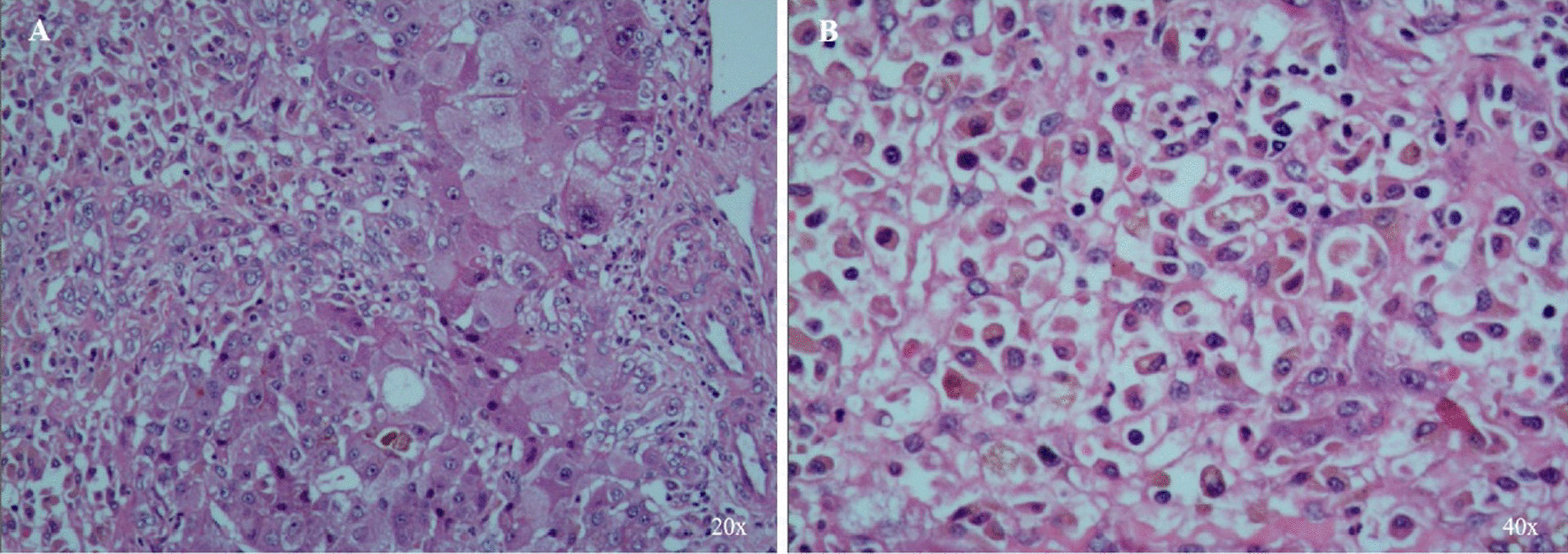
Postoperative pathology of liver under microscope. **A** Liver structure was damaged, with patchy necrosis, incomplete lobular structure, nest like or irregular scattered hepatocytes, hepatocyte necrosis and balloon like degeneration, liver juice stasis, disappearance of epithelial cells in the portal area and vitreous degeneration of vascular wall. There are many tissue cells in the stroma, accompanied by a small amount of inflammatory cell infiltration. Combined with medical history, it is consistent with toxic hepatitis and liver necrosis. **B** Lymphoid tissue of hilar lymph nodes hyperplasia, mainly sinus hyperplasia

On March 6, the patient was mentally normal. There were yellow spots on the skin with some bruises. Thrombocytopenia, prolonged coagulation function, decreased prothrombin time (PT), activated partial thromboplastin time (APTT) and plasma fibrinogen (FIB), increased transaminase and bilirubin were observed, Thus, the illness was diagnosed as toxic hepatitis. On March 7, the patient was mentally indifferent, with blood ammonia of 159.8 μmol/L and increased bilirubin and transaminase. From March 8 to March 10, the patient developed drowsiness into coma, with aggravated yellow skin and sclera. Besides, examinations found that bilirubin increased while transaminase decreased. The physiological and biochemical indexes of patient were as follows: alanine aminotransferase (ALT), 665 U/L; aspartate aminotransferase (AST), 197 U/L; total bilirubin, 170.8 μmol/L; direct bilirubin, 59.8 μmol/L; indirect bilirubin, 111 μmol/L; prothrombin time, 25.5 s; prothrombin time ratio (PTR), 2.13; activation time of partial thrombin time, 41 s; thrombin time, 21.5 s; fibrinogen quantification, 0.96 g/L; blood ammonia, 183.4 μmol/L. Moreover, the brain, chest and upper abdomen were scanned by computed tomography (CT): (1) the suspected parenchymal density decreased, and no intracerebral hemorrhage and obvious abnormality were observed; (2) both lungs (mainly lower lungs) were flaky with spot-like consolidation, which should be considered as inflammation; (3) the density of liver parenchyma decreased, and there was a small amount of effusion in abdominal cavity; (4) there was a cyst (45 mm × 44 mm) in right renal,, which suggested bleeding; (5) suspected pancreatic swelling and thickening of the anterior renal fascia were observed. However, the patient did not improve significantly after treatment and then developed into acute liver failure with toxic encephalopathy. Thereby, standard piggyback liver transplantation (SPBLT) was performed on March 10. The postoperative pathological changes of liver were consistent with toxic hepatitis and liver necrosis (Fig. 1A). In addition, hyperplasia was observed in hepatic hilar lymphoid tissue, mainly in the lymphatic sinuses (Fig. 1B). After the treatment of anti-infection, stomach protection, liver protection, removing jaundice, anti-rejection, adjustment of water and electrolyte balance, the patient’s consciousness gradually improved. A re-inspection was carried out on March 28, and the physiological and biochemical indexes (ALT, 160 U/L; direct bilirubin, 19.6 μmol/L; total bilirubin, 42.1 μmol/L) indicated that the coagulation disorder had been corrected. The mean portal vein velocity and hepatic artery resistance index were normal. Besides, there was fluid in the right chest whereas no obvious effusion was found in the abdominal cavity. Finally, the patient recovered and left hospital.

### Case 2

After routine medical treatment, the patient developed into a shallow coma. From March 11 to March 16, the condition of patient was gradually deteriorated and turned into coma. The physiological and biochemical indexes were as follows: blood ammonia, 118.7 μmol/L; ALT, 41 U/L; AST, 34 U/L; total bilirubin, 103.8 μmol/L; direct bilirubin, 52.9 μmol/L; indirect bilirubin, 50.9 μmol/L; prothrombin time, 19.5 s; prothrombin time ratio, 1.63; activated partial thromboplastin time, 42.1 s; thrombin time, 23.3 s; fibrinogen quantification, 1.01 g/L; platelet count, 38 × 10^9^/L. Besides, there was a small amount of pleural effusion and consolidation on the dorsal side of bilateral lower lungs. Both lungs showed patchy glass shadow, suggesting pulmonary edema. In addition, no obvious abnormalities were found in the brain. At this time, the patient with toxic acute liver failure combined disseminated intravascular coagulation (DIC) and hepatic encephalopathy were still treated mainly using medical treatment. Over the next 4 days, the patient’s consciousness was improved and treated by artificial extracorporeal liver support. On March 20, the patient suddenly felt confused and drowsy. The pupils dilated, and the reflection to light, corneal reflex, head eye reflex, and ciliary spine reflex disappeared. Besides, more pleural effusion and consolidation on dorsal side of bilateral lower lungs were observed. Upper left pneumonia absorbed more than before, pericardial effusion was about 13 mm. Moreover, pelvic effusion increased and there were muddy stones in the gallbladder. There was extensive subcutaneous edema in the abdominal wall. Furthermore, intracerebral hemorrhage occurred in the left occipital parietal lobe and entered the ventricle. The midline moved about 10 mm to the right. What’s worse, the patient had intracerebral hemorrhage and brain stem injury. Unfortunately, the patient died on March 26.

### Case 3

The patient was treated with routine medical treatment after admission. Results of chest X-ray showed pulmonary edema. On March 11, the patient was in a coma and continued to have a fever to 37.6 ℃. The sudden appearance of about 600 mL of blood in feces indicated that it was related to hemorrhagic enteritis, diffuse vascular coagulation and fatal amanitin vascular injury. Moreover, bilirubin increased while transaminase decreased, and platelets decreased gradually. Furthermore, the density of brain parenchyma decreased slightly, and the midline was in the middle, which were consistent with the changes of toxic encephalopathy. There was a small amount of pleural effusion on both sides. Both the right lower lung and the right upper lung were patchy nodules, suggesting pneumonia. Bilateral nephromegaly and multiple nodules of the right kidney need to be diagnosed. In addition, the uterus changed with pregnancy. The patients received intensive anti-infection, dehydration, improvement of brain edema, dynamic blood transfusion and other symptomatic treatment. On March 15, the patient was unconscious and still had fever (38.8 ℃). Besides, PCT, white blood cells (WBC), and the proportion of neutrophil (NE) increased significantly, blood ammonia decreased gradually, transaminase and coagulation function did not improve significantly, and AFP increased gradually. On March 20, the patient was conscious, and could open his eyes independently, and abdominal distension was aggravated. Moreover, the patient had pulmonary infection and extensive dilatation of the small intestine. Therefore, laxative treatment was carried out. On March 22, the patient was conscious and abdominal distension improved, however, the fever continued to 38.8 ℃. Besides, hematology and PCT decreased gradually, while bilirubin increased. Furthermore, there were fungal infections and intestinal obstruction. Thus, treatment programs such as anti-infection, blood purification and nutritional support were continued. On March 28, the patient was conscious, and continued to fever to 38.8 ℃. A miscarriage occurred in the early morning and the obstetrics department dealt accordingly. Moreover, the infection index was improved, the liver function was stable, and the coagulation function was also improved. The infection was identified as carbapenem resistant Acinetobacter baumannii, and the utilize of antibiotics was adjusted. On May 5, the patient was in a coma with different pupils and the bilateral nasolabial sulcus was symmetrical. Besides, the muscle tension of the left limb was low, and the left tendon reflex was weakened. Moreover, the left Babinski sign was positive, and the meningeal irritation sign was negative. The patient developed to massive hemorrhagic cerebral infarction and hernia. Mannitol was used to reduce intracranial pressure and right frontotemporal bone flap decompression was performed. However, the patient was still in a coma after the operation. On May 15, the patient’s family requested to leave hospital.

## Discussion and conclusion

Amanita poisoning may initiate liver injury, which is the main cause of death by wild mushroom poisoning. The degree of liver damage is positively correlated with mushroom intake [8]. Its pathogenesis can be divided into six periods including incubation period, gastroenteritis period, false healing period, organ injury period, mental symptom period and recovery period [9]. After being absorbed by intestinal epithelium, toxoid mainly acts on liver nucleus, inhibits RNA polymerase and reduces liver glycogen synthesis [10]. In addition, about 60% of toxins are excreted into bile and return to the liver through hepato-intestinal circulation, resulting in toxic inflammation and hepatocyte necrosis [11].

“False healing period” is a characteristic of poisonous mushroom poisoning, that is, vomiting, diarrhea and other symptoms occur after the poisoning is improved, resulting in patients ignoring medical treatment [12]. Elimination of gastrointestinal toxicants and protection of liver and kidney are the main prerequisites for successful treatment [13]. In order to prevent the gastrointestinal tract from absorbing toxins in the early stage of the disease and promote the clearance of toxins in the systemic circulation, comprehensive multidisciplinary treatment should be adopted [14]. With the deterioration of coagulation dysfunction and bile enzyme separation, acute liver failure is prone to occur [15]. At this time, drug treatment cannot improve poisoning symptoms, poor prognosis and high mortality. According to the Chinese guidelines for clinical diagnosis and treatment of mushroom poisoning, the common treatment methods are as follows. Blood purification therapy can improve the clearance rate of poisons and support organ function. It has been widely used in the treatment of mushroom poisoning [16]. Besides, patients with mushroom poisoning, especially those with amanitin related mushroom poisoning, should be treated with detoxifying drugs, such as penicillin G, silymarin, *N*-acetylcysteine (NAC), Ganoderma lucidum, and sulfhydryl compounds [17]. Moreover, organ function support therapy can prevent infection, support nutrition, and maintain the balance of water and electrolyte [18]. Liver transplantation is the last treatment for liver failure caused by mushroom poisoning [19]. However, there are no clear guidelines for liver transplantation for acute liver failure caused by mushroom poisoning.

There is no specific antidote to this mushroom poisoning. The basic treatments include drug therapy, artificial liver support therapy and surgical liver transplantation [20]. In this study, three patients received comprehensive treatment immediately after admission. Case 1 was treated with drugs and continuous artificial liver. However, the liver function did not improve and further developed into stage IV hepatic encephalopathy and acute liver failure. Therefore, we urgently carried out liver transplantation. For acute liver failure caused by mushroom poisoning, there is no clear guideline for liver transplantation. According to the judgment criteria of King’s college, Ganzert’s, Escudié’s and MELD scores [21] (Table 3), many patients received liver transplantation and successfully treat toxic hepatitis and liver failure caused by mushrooms. As reported by Yantorno et al. [22], for patients with non-acetaminophen induced acute liver failure, if the MELD score is higher than 30 and there is no emergency liver transplantation, the mortality was as high as 94%. In addition, Cox model analysis shows that the statistical value of MELD integral was much higher than the above foreign standards. MELD score is helpful to predict the risk of acute liver failure and judge the timing of liver transplantation. In this study, the MELD score of case 1 was 36. Although the preoperative examination results did not meet the above three transplantation criteria, we chose reasonable indications, excluded contraindications, grasped the operation opportunity, effectively treated liver failure and improved its prognosis through liver transplantation.

**Table 3:** Commonly foreign standards for liver transplantation for acute liver failure patients

King’s college criteria	Ganzert’s criteria	Escudié’s criteria
PT > 100 s (INR > 6.5) or meet the following three requirements: A: Age < 10 years or > 40 years B: Jaundice duration (before hepatic encephalopathy) > 7 days C: PT > 50 s(INR > 3.5) D: serum bilirubin > 300 μmol/L E: Etiology: non-A and B hepatitis, flurane induced hepatitis, specific drug-induced hepatitis	a: Prothrombin activity < 25% in 3–10 days of ingestion b: When PT was prolonged, serum creatinine was ≥ 10^6^ μ/L (1.2 mg/dL)	a: The interval between intake and diarrhea is less than 8 h Or b: prothrombin activity < 10% from 4th day after ingestion

Brain edema caused by intracerebral hemorrhage is the main cause of death in patients with acute liver failure. Severe irreversible brain injury is considered to be one of the relative contraindications of liver transplantation [23]. Previous studies have shown that patients with acute liver failure complicated with cerebrovascular disease after liver transplantation had lower 1-year survival rate and higher postoperative complications [24]. Cognitive impairment and vegetative state seriously affect the quality of life of patients. Cases 2 and 3 had false healing period, and the improvement of consciousness often misled the medical staff for treatment. After active and conservative treatment, liver function and coagulation function were improved, followed by large-area intraventricular hemorrhage, indicating a poor prognosis and liver transplantation can reverse the symptoms of poisoning. The choice of patients, the judgment of transplantation time and the shortage of organ supply seriously affected the prognosis. Premature liver transplantation may lead to unnecessary surgery and waste of liver resources, while late liver transplantation may lead to complications such as multiple organ failure, loss of surgical opportunities or poor prognosis [25]. In this research, the diagnosis and treatment of three patients were studied. For patients whose liver function cannot be improved after drug treatment, hepatic encephalopathy stage III–IV or MELD score is higher than 30, it was recommended to start liver transplantation immediately to avoid missing the best operation opportunity. However, the number of cases in this study is very small. With the improvement of various indexes, whether to carry out emergency liver transplantation needs to accumulate clinical data.

In conclusion, there is no internationally recognized liver transplantation guideline for liver failure caused by mushroom poisoning. Although King’s college, Ganzert’s, Escudié’s and MELD scores have important reference significance in the evaluation of prognosis, selection of surgical indications and timing of operation in patients with acute liver failure, there are still deficiencies. More research is needed to further clarify the indications and opportunities of liver transplantation in these cases, and jointly develop unified guidelines to improve the survival rate of patients.

## Data Availability

The datasets used and analyzed during the current study are available from the corresponding author on reasonable request.

## Abbreviations

ALT: Alanine aminotransferase
APTT: Activated partial thromboplastin time
AST: Aspartate aminotransferase
CT: Computed tomography
DIC: Disseminated intravascular coagulation
FIB: Plasma fibrinogen
NAC: *N*-Acetylcysteine
NE: Proportion of neutrophil
PT: Prothrombin time
PTR: Prothrombin time ratio
SPBLT: Standard piggyback liver transplantation
WBC: White blood cells
